# The flipped classroom in an undergraduate nutritional science course: A pilot study

**DOI:** 10.1186/2193-1801-4-S2-P3

**Published:** 2015-11-27

**Authors:** Sidney Man-ngai Chan, Jean Yuk-tin Tse, Peter Hoi-fu Yu

**Affiliations:** Faculty of Science and Technology, Technological and Higher Education Institute of Hong Kong, Hong Kong; School of Science and Technology, The Open University of Hong Kong, Hong Kong; School of General Education and Languages, Technological and Higher Education Institute of Hong Kong, Hong Kong

## Background

The flipped classroom pedagogy has gained popularity over the past decade as an alternative to more traditional lecture-style classes. This teaching technique typically involves students previewing lecture materials before class and participating in classroom activities designed so that they can demonstrate understanding. By 'flipping' a class, i.e., reversing school and home activities, teachers can look more closely at the difficulties experienced by their students and offer help where and when it is needed.

## Methods

A randomised control trial was conducted to evaluate the flipped classroom pedagogy for a freshman year nutritional science module. While the control group (N = 18) received traditional lecturing in nine weekly sessions, the experimental group (N = 20) was given access to additional resources on Moodle ahead of each session: video lectures of two to eight minutes in length and quizzes composed of short, close-ended items. This experimental group also engaged in interactive activities during class sessions.

Before the start and at the end of the module both groups were given questionnaires designed to elicit opinions about the two versions of the module and to gauge the students' attitudes towards learning in general and flipped learning.

## Results

The flipped classroom in the present study was a partial kind, in which only a fraction of each module topic was delivered through video lectures. Half the experimental group attempted the Moodle quiz prior to class more than half the time. Overall this group's preference for flipped learning was positively correlated with whether or not the students thought the video lectures were interesting and whether or not they liked to participate in activities that were connected to the topic at hand.

Compared to the control group, the experimental group rated the Moodle platform more highly when it came to helping them manage their progress in this module. Those students were also more likely to seek help from the teacher when they had difficulties. However, when a mid-term exercise was analysed for differences in performance between the control and the experimental groups no statistical difference was found.

## Conclusions

The present study demonstrated the use of the flipped classroom pedagogy in the context of post-secondary nutrition education. The pedagogy was positively received in general, which indicates its potential to enhance learning experiences in vocational education and training.
